# Infrapatellar Fat Pad: An Alternative Source of Adipose-Derived Mesenchymal Stem Cells

**DOI:** 10.1155/2016/4019873

**Published:** 2016-04-26

**Authors:** P. Tangchitphisut, N. Srikaew, S. Numhom, A. Tangprasittipap, P. Woratanarat, S. Wongsak, C. Kijkunasathian, S. Hongeng, I. R. Murray, T. Tawonsawatruk

**Affiliations:** ^1^Department of Orthopaedics, Faculty of Medicine, Ramathibodi Hospital, Bangkok 10400, Thailand; ^2^Office of Research and Innovation, Ramathibodi Hospital, Bangkok 10400, Thailand; ^3^Department of Surgery, Faculty of Medicine, Ramathibodi Hospital, Bangkok 10400, Thailand; ^4^Department of Pediatrics, Faculty of Medicine, Ramathibodi Hospital, Bangkok 10400, Thailand; ^5^Department of Orthopaedics, Royal Infirmary Edinburgh Hospital, Edinburgh University, Edinburgh, UK

## Abstract

*Introduction*. The Infrapatellar fat pad (IPFP) represents an emerging alternative source of adipose-derived mesenchymal stem cells (ASCs). We compared the characteristics and differentiation capacity of ASCs isolated from IPFP and SC.* Materials and Methods*. ASCs were harvested from either IPFP or SC. IPFPs were collected from patients undergoing total knee arthroplasty (TKA), whereas subcutaneous tissues were collected from patients undergoing lipoaspiration. Immunophenotypes of surface antigens were evaluated. Their ability to form colony-forming units (CFUs) and their differentiation potential were determined. The ASCs karyotype was evaluated.* Results*. There was no difference in the number of CFUs and size of CFUs between IPFP and SC sources. ASCs isolated from both sources had a normal karyotype. The mesenchymal stem cells (MSCs) markers on flow cytometry was equivalent. IPFP-ASCs demonstrated significantly higher expression of SOX-9 and RUNX-2 over ASCs isolated from SC (6.19 ± 5.56-, 0.47 ± 0.62-fold; *p* value = 0.047, and 17.33 ± 10.80-, 1.56 ± 1.31-fold; *p* value = 0.030, resp.).* Discussion and Conclusion*. CFU assay of IPFP-ASCs and SC-ASCs harvested by lipoaspiration technique was equivalent. The expression of key chondrogenic and osteogenic genes was increased in cells isolated from IPFP. IPFP should be considered a high quality alternative source of ASCs.

## 1. Introduction

Thailand has a rapidly ageing society. The population aged over 65 years has grown markedly over the last decade [1]. According to a report by the Institute for Population and Social Research, over 9 million people were considered elderly in Thailand in 2015, with this increasing by 7 percent per year over the coming 5 years [1]. As a result, there will be a parallel increase in degenerative diseases. Osteoarthritis (OA) is the most common cause of joint diseases [2] with the knee most commonly affected joint (approximately 50.2 percent) [3]. Progression of OA can result in severe disability, compromising quality of life [4] and having significant financial implications for individuals and society. There are currently no treatments that effectively regenerate cartilage or delay the progression of OA.

Mesenchymal stem cells (MSCs) derived from bone marrow have been evaluated in the treatment of OA [5]. A number of studies have demonstrated that MSCs can be isolated from multiple tissue sources including blood, bone marrow, adipose tissue, and cartilage [6]. MSCs have the ability to differentiate into adipocytes, osteocytes, and chondrocytes, while contributing to a regenerative microenvironment through the release of trophic factors [7]. As such, MSC based approaches may be promising in the treatment of osteoarthritis. However, there have so far been few well-designed randomized controlled trials [8, 9], with many existing studies evaluating low patient numbers with incomplete treatment evaluation. Further studies are required before the widespread application of MSCs in clinical practice. Furthermore, there is ongoing controversy relating to optimizing cell source, cell preparation conditions, ideal dosing, and method of delivery [5, 10–13].

Cell source is one factor that may influence treatment outcomes. It has been reported that adipose-derived mesenchymal stem cells (ASCs) had more rapid cell doubling times and antiapoptotic potential when compared to MSCs from other sources [14]. There have been an increasing number of studies evaluating ASCs in both experimental and clinical studies in over the past 10–20 years. Overall these studies have demonstrated promising results for ASCs in the management of OA knee and chondral injury [5, 10–12, 15–19]. Underlying mechanisms have been elucidated including the release of various cytokines from MSCs that resulted in anti-inflammatory effects and increased regeneration of damaged cells [20–24]. MSCs have also resulted in improved clinical outcomes including knee motion, decreased visual analog pain scale (VAS), and improved quality of life as evaluated by the WOMAC index. Stem cell therapy for knee OA has been found to be safe with no major complications or adverse effects reported in treatments to date [5, 8, 10–12].

The infrapatellar fat pad (IPFP) is located in the knee joint [25, 26]. IPFP can be easily resected during knee surgery either open or arthroscopically [27]. The isolation of MSCs has previously been reported from this source [28]. However, there are a few studies comparing the characteristics and differentiation potentials between ASCs isolated from IPFP and subcutaneous tissue (SC) [9, 29, 30].

Therefore, this study aimed to evaluate (1) the characteristics and (2) differentiation abilities of ASCs isolated from different sources (IPFP versus SC). The safety profiles, including bacterial infection and karyotype, were also examined. Ultimately this study sought to identify the most appropriate source of ASCs for cell-based treatment of OA knee.

## 2. Materials and Methods

### 2.1. Study Design and Patients

This study was designed as an* in vitro* experimental study and conducted between December 2014 and April 2015 at Ramathibodi Hospital, Bangkok, Thailand. It was approved by Committee on Human Rights Related to Research Involving Human Subjects, Faculty of Medicine, Ramathibodi Hospital (MURA2014/476). All participants provided informed consent before being enrolled to the study. Five patients aged over 50 years diagnosed with primary OA (Kellgren-Lawrence (KL) classification 3 or 4) scheduled for total knee arthroplasty (TKA) were included in this study for IPFP harvesting. 5 samples of SC were collected from patients who underwent cosmetic liposuction.

### 2.2. Infrapatellar Fat Pad Adipose-Derived Mesenchymal Stem Cells (IPFP-ASCs) Isolation and Culture Technique

IPFPs were harvested during TKA with tissues and then used for cell isolation. In brief, IPFP tissues were washed with phosphate buffer solution (PBS), rinsed, and then chopped in small pieces of about 0.5 mm^3^. Tissues were washed again in 5 mL of PBS two further times. 0.1% collagenase type I (Gibco®, USA) was added and incubated under warm-water bath (37°C) for 60 minutes then suspended with Dulbecco's Modified Eagle Medium-Low Glucose (DMEM-LG; Biochrom®, Germany) with 10% fetal bovine serum (FBS; Biochrom, Germany) and centrifuged under 400x for 10 minutes and surfactant layer was removed. The retained pellet was mixed with 10 mL of PBS, then passed through a sterile filter (Cell Strainer®), and centrifuged at the same speed. The cell pellet was resuspended in 1 mL of complete medium (DMEM-LG + 10% FBS + 1% L-glutamic acid (Gibco, USA) + 1% Penstrep (Gibco, USA) + 0.1% Amphotericin-B (Fungizone®)) and then divided for cell counting using a hemocytometer. Cells were then seeded in T-25 tissue culture flasks at a density of 5,000 cell/cm^2^ at 37°C (humidified atmosphere 95% O_2_ and 5% CO_2_). The medium was changed every 2 days. On days 7–10, the cells were accessed and microscopic appearance was observed by at least 2 investigators. If cells covered more than 80% of the culture flask, then ASCs were detached with 0.05% Trypsin/0.1% EDTA (Gibco, Canada) and recultured as the first passage with complete medium through 3rd passage. Cell count and time between each passage were recorded. MSCs in 3rd passage were trypsinized and divided for cell phenotypes, safety profiles, and ASCs differentiation assays. SC was processed as per IPFP for cell isolation and culture expansion.

### 2.3. ASCs Immunophenotypes and Safety Profiles Assessment

Positive cell surface markers (CD 73, CD 90, and CD 105; eBioscience®, USA) and negative cell surface markers (CD 34, CD 45, and HLA-DR; eBioscience, USA) were evaluated by flow cytometry.

Individual samples of 1 × 10^5^ cells of ASCs from each patient were resuspended in 3 mL PBS that was sent to Central Laboratory, Department of Pathology, Ramathibodi Hospital, for evaluation of aerobe/anaerobe bacterium, mycobacterium, and mycoplasma contamination.

For ASCs karyotype assessment, complete medium was aspirated from 3rd passage of T-25 flask and 10 *μ*L/mL Colcemic solution (Gibco, USA) added and incubated for 3.5 hrs to inhibit the cell cycle. 5 mLs of PBS solution was added to wash out Colcemic solution prior to trypsinization centrifuge pelleting. The pellet was resuspended in 10 mL of 0.068 M potassium chloride (KCl) solution prior to incubation for 15 min with gentle cyclic inversion of the tube. Finally, samples were sent to Genetic Section, Ramathibodi Hospital, for cell karyotype assessment. At least 2 metaphase cells were compared for each set of homologues chromosomes manually [31].

### 2.4. Colony-Forming Unit (CFU) Assay

ASCs from both SC and IPFP were seeded in polypropylene 6-well plates, which 3 different dilutions (100, 250, and 500 cells/well) to evaluated ASCs proliferation potential. Cells were cultured under complete media for 14 days; then cells were fixed by methanol and stained with Giemsa solution. The number of colonies was then counted under a light microscope (4x). The largest colonies in each quarter of a culture well were captured under 4x microscope and the size of colonies was counted in duplicate manner using ImageJ software version 1.48 (NIH, USA) [32].

### 2.5. ASCs Differentiation Abilities Assessment

Chondrogenic, adipogenic, and osteogenic differentiation potential were determined. 5 × 10^6^ cells from 3rd passage of IPFP-ASCs and subcutaneous adipose-derived mesenchymal stem cells (SC-ASCs) were split and cultured under specific medium T-25 flask for adipogenic and osteogenic differentiation but cultured in 6-well plate for chondrogenic differentiation. Time for ASCs culture in specific medium was 21 days.

For chondrogenic differentiation medium, we used commercial medium from PromoCell®, USA. Adipogenic and osteogenic differentiation media were combined with Dulbecco's Modified Eagle Medium-Low Glucose (DMEM-LG), Ham's-12, 10% FBS, 1% L-glutamic acid, and 1% Penstrep. Isobutyl methylxanthine (IBMX; Sigma, Japan), Dexamethasone (Sigma, Japan), Insulin (Sigma, Japan), and Indomethacin (Sigma, China) with based medium were used for adipogenic differentiation medium. Also, Dexamethasone, *β*-glycerophosphate (Sigma, USA), and Ascorbic Acid (Sigma, Japan) were combined with based medium for osteogenic differentiation medium.

At day 21, cells were stained to identify cell morphology. Chondrogenic-induced ASCs were stained by Alcian Blue staining solution (staining dark blue for aggrecan). Osteogenic-induced ASCs were stained using Alizarin Red staining solution that detected extracellular calcium deposition (bright orange-red). Adipogenic-induced ASCs were stained using Oil Red-O (intracellular lipid vesicles staining bright red).

Furthermore, RNA extraction by Trizol® technique and cDNA synthesis (RealMasterScript*™*, SuperMix Kit) were performed. Real-time polymerase chain (RT-PCR, LightCycler® 480 II) was performed for quantitative assessment of SOX-9, RUNX-2, and PPAR-*γ* expression that were represented for chondrogenic, osteogenic, and adipogenic differentiation. Primer sequences of SOX-9, RUNX-2, PPAR-*γ*, and GAPDH are showed in Table 1.

### 2.6. Statistical Analysis

Overall 10 ASCs samples (5 samples from IPFP and the others from SC) were analyzed by SPPS version 15.0 (IBM, USA). A *p* value < 0.05 was considered significant in this study. Analysis of variance was calculated for data comparison in demographic data of patients, MSCs immunophenotypes, CFUs, and gene expression of ASCs in each source.

## 3. Results

### 3.1. Demographic Data

IPFPs were collected by sterile technique from 5 female participants undergoing TKA. Patient's age ranged from 53 to 77 years, with BMI ranging from 20.24 to 26.53 kg/m^2^. All participants were diagnosed with OA right knee stage 4 by KL classification. The IPFP was measured for weight (range 8.48–14.75 g) and for yield for cell extraction (range 7.88 × 10^4^–67.79 × 10^4^ cell/g). Mean time for cell culture from 0th passage to 2nd passage was 18.40 ± 5.50 days (range 14–28 days) (Table 2).

The baseline characteristics of SC participants are showed in Table 3. Patient's age ranged from 16 to 48 years, with BMI ranging from 17.70 to 36.29 kg/m^2^. SC tissue was measured for weight (range 28.30–75.85 g) and for yield for cell extraction (range 3.49 × 10^4^–21.76 × 10^4^ cell/g). Mean time for cell culture from 0th passage to 2nd passage was 19.80 ± 8.04 days (range 14–33 days).

In comparison of ASC properties between IPFP and SC source, this study found nonstatistical significance in gender and BMI of participants both groups (*p* value = 1.000 and *p* value = 0.953, resp.) but the mean age in SC group was lower than IPFP group (*p* value = 0.001). The weight of adipose tissue from lipoaspiration was more than TKA operation (52.46 ± 21.62, 12.12 ± 2.57 g; *p* value = 0.014). The number of ASCs isolated from each source was not statistically different (*p* value = 0.602) but IPFP group had significantly higher yield of ASCs collection than other groups (33.39 ± 30.54 and 8.94 ± 7.34; *p* value = 0.047). There was no statistically significant difference in the time for ASC cultures to reach 2nd passage between groups (*p* value = 0.833), as shown in Table 4.

### 3.2. ASCs Immunophenotypes

**Figure 1:**
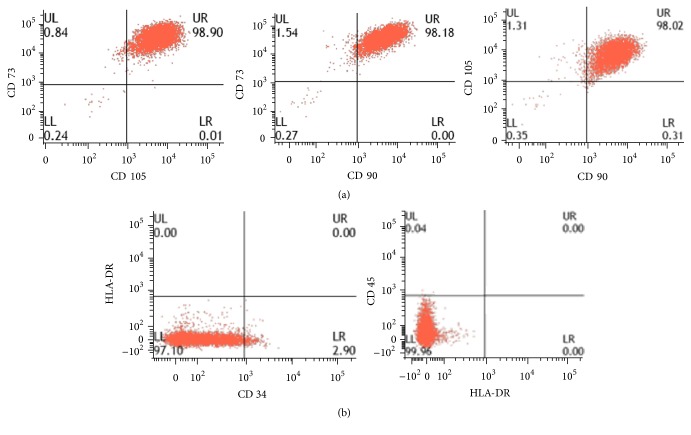
Flow cytometry of IPFP-ASCs (Case number 4). First row showed positive markers (CD 90, CD 105, and CD 73) and second row showed negative markers (CD 34, CD 45, and HLA-DR).

The phenotype of ASCs was similar between groups as assessed by flow cytometry. Positive markers for ASCs were shown by CD 73, CD 90, and CD 105. Negative markers for ASCs were shown by CD 34, CD 45, and HLA-DR (Table 3 and Figure 1).

### 3.3. Colony-Forming Unit Evaluation

**Figure 2:**
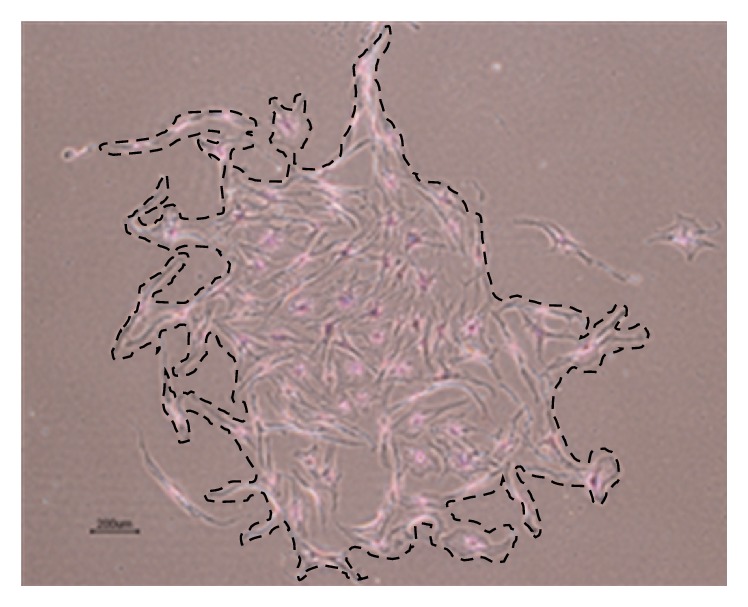
CFU of IPFP-ASCs (Case number 4) under light microscope (magnifier 4x). Dashed line was drawn along border of CFU for size calculation by ImageJ software.

CFU counting was performed after 14 days of culture in polypropylene 6-well plates (Figure 2). IPFP-ASCs had fewer CFUs (3.13 ± 1.71 and 3.99 ± 1.52 CFUs per 100 cells) which were smaller than SC-ASCs (9.91 ± 4.72 and 12.99 ± 3.26 mm^2^) but this did not reach statistical significance (*p* value = 0.428 and 0.263, resp.), as showed in Table 4.

### 3.4. Safety Assessment

**Figure 3:**
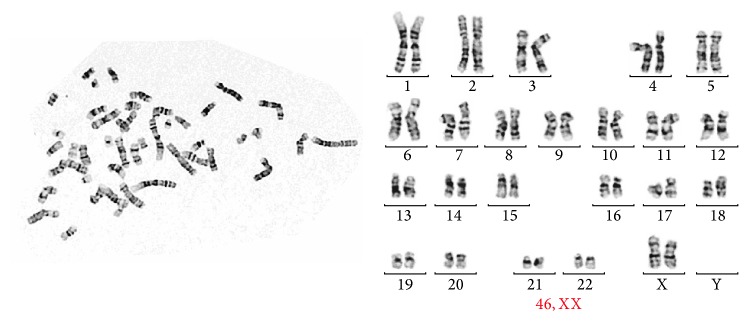
Normal karyotype of IPFP-ASCs (46, XX).

There was no evidence of bacterial or other pathogen contaminations in culture. PCR for mycobacterium and mycoplasma detection was all negative. Normal cell karyotypes were evaluated (Figure 3).

### 3.5. ASCs Differentiation and Histology

**Figure 4:**
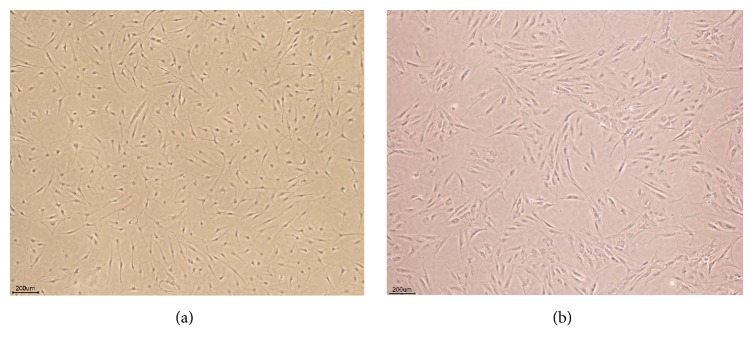
(a) IPFP-ASCs and (b) SC-ASCs morphology at 7th day of 2nd passage under light microscope (4x).

**Figure 5 fig5:**
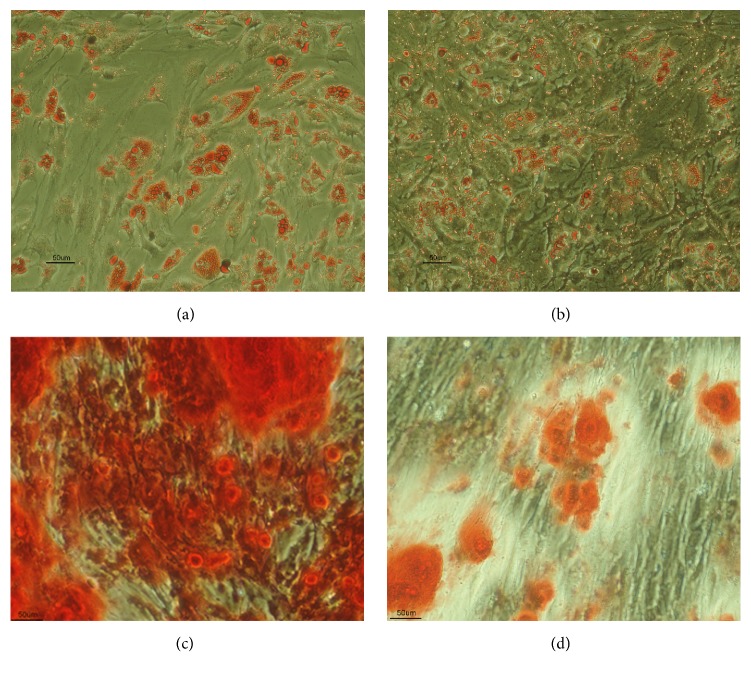
ASCs differentiation to adipocyte from IPFP (a) and SC (b) which stained by Oil Red-O (20x). Osteogenic differentiation to from IPFP (c) and SC (d) which staining by Alizarin Red (20x).

**Figure 6:**
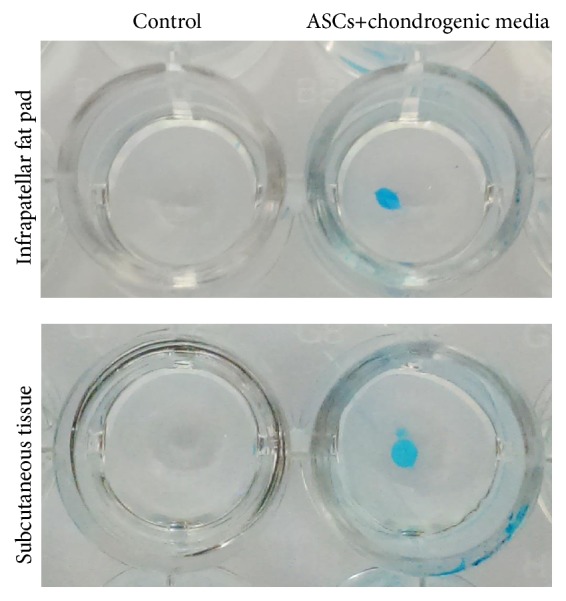
ASCs differentiation to chondrocyte from IPFP and SC which stained by Alcian Blue. Both groups showed blue staining of the spheroid of chondrocyte.

The morphology of ASCs was assessed using light microscopy prior to induction of differentiation. ASCs appeared as flat polygonal cells. Following differentiation cells were stained using appropriate specific staining solutions with positive staining seen following osteogenic, adipogenic, and chondrogenic differentiation (Figures 4 [Fig fig5]–6).

### 3.6. Gene Expression of ASCs

**Figure 7:**
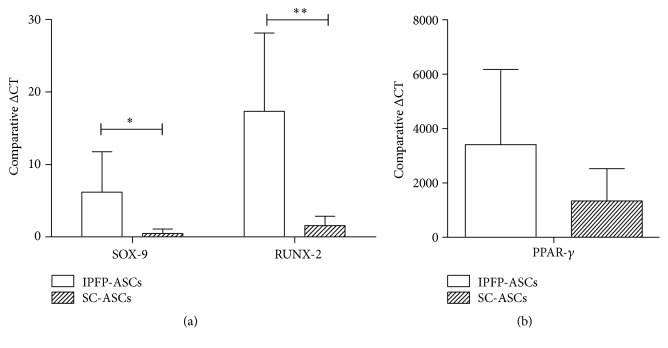
(a) Comparison of SOX-9 and RUNX-2 expression in IPFP-ASCs and SC-ASCs. (b) Comparison of PPAR-*γ* expression in IPFP-ASCs and SC-ASCs. ^*∗*^ *p*-value < 0.05 and ^*∗∗*^ *p*-value < 0.01.

IPFP-ASCs showed significantly higher expression of SOX-9 (6.19 ± 5.56- and 0.47 ± 0.62-fold; *p* value = 0.047) and RUNX-2 (17.33 ± 10.80- and 1.56 ± 1.31-fold; *p* value = 0.030) than SC-ASCs using the ΔCT formula (2^−ΔΔCT^) (Figure 7(a)). Furthermore, the expression of PPAR-*γ* by IPFP-ASCs was not significantly higher than SMSCs (3,404.40 ± 2,763.47- and 1,335.33 ± 1,190.28-fold; *p* value = 0.163) (Figure 7(b)).

## 4. Discussion

The implantation of MSCs holds great promise as a strategy to improve bone and cartilage regeneration. The “cellular substrate” represents one aspect of the tissue engineering triad, with MSCs considered the principal progenitors of several musculoskeletal tissues. It has been reported that this cell type can be isolated from several tissues [6]. Adipose tissue is more accessible than bone marrow. However, the previous studies have demonstrated that there are considerable differences in growth and differentiation between different sources of MSCs [33, 34]. Because of the accessibility of adipose tissue, further investigation should focus on how this cell type might be utilized effectively. Moreover, MSCs derived from adipose tissues have been shown to be more effective in their multipotent potential compared to other sources. ASCs had low population doubling time for cell proliferation and higher antiapoptosis potential than mesenchymal cells derived from bone marrow and cartilage [14].

This study focused on ASCs isolated from 2 different sources, namely, subcutaneous tissue (SC) and infrapatellar fat pad (IPFP). ASCs' sterility and karyotype were evaluated in this study as safety is a key consideration in regenerative therapies. There was no contamination during the process of cell isolation and culture expansion. With respect to general morphology, cells isolated from both sources exhibited a fibroblast liked appearance and plasticity without significant differences. This study found that IPFP-ASCs and SC-ASCs had similar cell surface profiles demonstrated by flow cytometry including positive expression of CD 73, CD 90, and CD 105 and negative expression of CD 34, CD 45, and HLA-DR. Their proliferation potentials were evaluated using clonogenic assay by number and size of CFUs. We observed no differences between both groups. With respect to differentiation ability, IPFP-ASCs showed superiority in osteogenic and chondrogenic differentiation over SC-ASCs as assessed by SOX-9 and RUNX-2 expression representing chondrogenic differentiation and osteogenic differentiation, respectively. Thus, we conclude from this* in vitro* study that the infrapatellar fat pad may be a good candidate source for cartilage and bone regeneration.

The IPFP is an intracapsular and extrasynovial store of adipose tissue located between the patellar tendon, the femoral condyle, and the tibial plateau [25]. The IPFP has previously been identified as a minimally invasive and easily accessible source of ASCs that can be obtained safely by orthopaedic surgeons using an arthroscopic approach with minimal morbidity [28, 35]. Conventionally, subcutaneous fat has been obtained using lipoaspiration with a key advantage being the large volumes of tissue that can be obtained in this way. However, lipoaspiration is associated with complications such as scarring, surface irregularities or skin necrosis, seroma, hematoma, allergic reactions to drugs, skin discoloration, temporary bruising, numbness or nerve injury, and temporary adverse drug reaction [36]. Although the total number of cells harvested from IPFP was more than SC in this study, when normalized by tissue weight, the number of ASCs isolated from the IPFP was significantly higher than from SC.

Anatomical area from which tissue is isolated may affect gene expression. IPFP is in close contact with synovial membrane and synovial fluid; this microenvironment may affect the characteristics of contained ASCs. Bobacz et al. demonstrated that growth differentiation factor 5 (GDF-5 or cartilage derived morphogenic protein 1) and BMP-7 (osteogenic protein 1) were expressed in higher levels in synovial tissue than articular cartilage [37] potentially increasing the chondrogenic differentiation of IPFP-ASCs. Our findings that SOX-9, RUNX-2, and PPAR-*γ* expression is higher in IPFP-ASCs than SC-ASCs is in keeping with Lopa et al. These authors found that IPFP-ASCs had lower expression of the hypertrophic and fibrogenic markers COL10A1 and COL1A1 [29]. As a result, we feel that IPFP-ASCs may provide a cellular substrate capable of high quality chondrogenesis in cartilage therapy.

A limitation of the present study was a lack of paired samples of IPFP and SC from individual donors. This was not possible due to ethical considerations. Differences in age between groups may have influenced the biological characteristics of tissue and cells. Previous studies have not found any influence of gender on ASCs characteristics by expression of cell surface markers such as CD 34, CD 44, CD 45, CD 54, CD 73, CD 80, CD 90, CD 105, CD 106, and CD 166 [38]. Similarly, Scharstuhl et al. reported that there was no correlation between mRNA expression for types 1 and 2 collagen and donor age in bone marrow-derived mesenchymal stem cell from 98 osteoarthritis patients [39]. Choudhery et al. reported no difference between adipogenic (PPAR-*γ* and lipoprotein lipase) and neurogenic (neurofilament and neuron-specific-enolase) gene expression of SC-ASCs compared in patients from groups of different ages [40]. Khan et al. reported that there was no difference in osteogenic differentiation potential of IPFP-ASCs and expression of alkaline phosphatase and osteocalcin expression between patient groups: mean age 57 ± 3 years group and 86 ± 3 years group [41].

Accordingly, ASCs from the infrapatellar fat pad may be considered good alternative source of ASCs because of their expression of chondrogenic and osteogenic genes. Further investigations in which IPFP-ASCs are evaluated in an animal model setting are now required to evaluate clinical outcomes such as quality of IPFP-ASCs induced cartilage and cartilage transplantation technique.

## 5. Conclusion

IPFP represents a good alternative source for MSCs to conventional SC-derived cells. General characteristics were similar between IPFP-ASCs and SC-ASCs but gene expressions for differentiation to a chondrogenic and osteogenic lineage were higher within IPFP-derived cells. IPFP should be considered a high quality source for bone and cartilage regeneration therapy.

## Figures and Tables

**Table 1:** Primer sequences.

Gene	Sequence forward	Sequence reverse
SOX-9	5′AGGTGCTCAAAGGCTACGAC 3′	5′ GTAATCCGGGTGGTCCTTCT 3′
RUNX-2	5′ CGGAATGCCTCTGCTGTTAT 3′	5′ TTCCCGAGGTCCATCTACTG 3′
PPAR-*γ*	5′ CCAGAAAGCGATTCCTTCAC 3′	5′ TGCAACCACTGGATCTGTTC 3′
GAPDH	5′ TGTTGCCATCAATGACCCCTT 3′	5′ CTCCACGACGTACTCAGCG 3′

**Table 2:** Baseline characteristics of IPFP-ASCs.

	Case 1	Case 2	Case 3	Case 4	Case 5
Sex	Female	Female	Female	Female	Female
Age (yrs)	77	53	71	66	62
BMI (kg/m^2^)	26.53	27.01	24.44	20.50	20.24
Side of TKA operation	Right	Right	Right	Right	Right
OA classification	Stage 4	Stage 4	Stage 4	Stage 4	Stage 4
Fat pad weight (g)	13.49	8.48	10.25	8.63	14.75
Number of ASCs isolation (P0^*∗*^) (×10^6^ cells)	9.10	1.25	6.75	1.43	1.16
Yield of ASCs collection (P0^*∗*^) (×10^4^ cells/g)	67.46	9.27	67.79	16.51	7.88
Incubation time (days)	17	28	17	16	14
P0 to P1^*∗∗*^	7	10	10	6	7
P1 to P2^*∗∗∗*^	10	18	7	10	7

^*∗*^0th passage.

^*∗∗*^1st passage.

^*∗∗∗*^2nd passage.

**Table 3:** Baseline characteristics of SC-ASCs.

	Case 1	Case 2	Case 3	Case 4	Case 5
Sex	Male	Female	Female	Female	Female
Age (yrs)	32	16	19	48	37
BMI (kg/m^2^)	17.70	25.65	19.91	36.29	18.03
Fat pad weight (g)	28.30	64.43	63.18	75.85	30.55
Number of ASCs isolation (P0^*∗*^) (×10^6^ cells)	2.20	4.10	3.35	2.65	6.65
Yield of ASCs collection (P0^*∗*^) (×10^4^ cells/g)	7.78	8.06	5.30	3.49	21.76
Incubation time (days)	22	14	15	33	15
P0 to P1^*∗∗*^	7	7	8	19	7
P1 to P2^*∗∗∗*^	15	7	7	14	8

^*∗*^0th passage.

^*∗∗*^1st passage.

^*∗∗∗*^2nd passage.

**Table 4:** Comparison of baseline characteristics between IPFP-ASCs and SC-ASCs.

	Infrapatellar fat pad (*N* = 5) *n* (%)	Subcutaneous (*N* = 5) *n* (%)	*p* value
Gender			1.000^†^
Male	—	1 (20.00)	
Female	5 (100.00)	4 (80.00)	
Age (yrs)^*∗*^	65.80 ± 9.09	30.40 ± 13.16	**0.001**
BMI (kg/m^2^)^*∗*^	23.75 ± 3.23	23.52 ± 7.82	0.953
Weight of fat collection (g)^*∗*^	12.12 ± 2.57	52.46 ± 21.62	**0.014**
Number of ASCs isolation (P0) (×10^6^ cells)^*∗*^	3.94 ± 3.73	3.79 ± 1.75	0.602^‡^
Number of ASCs (P0) per weight (×10^4^ cells/g)^*∗*^	33.39 ± 30.54	8.94 ± 7.34	**0.047** ^‡^
Incubation time to P2 (days)^*∗*^	18.40 ± 5.50	19.80 ± 8.04	0.833^‡^
ASCs markers			
Positive markers			
CD 73 (%)^*∗*^	99.69 ± 0.26	99.60 ± 0.26	0.591
CD 90 (%)^*∗*^	91.43 ± 10.79	93.85 ± 6.71	0.917^‡^
CD 105 (%)^*∗*^	90.63 ± 9.49	87.85 ± 19.79	0.917^‡^
Negative markers			
CD 34 (%)^*∗*^	1.24 ± 1.54	1.46 ± 2.22	0.917^‡^
CD 45 (%)^*∗*^	0.17 ± 0.08	0.16 ± 0.12	0.882
HLA-DR (%)^*∗*^	0.55 ± 0.69	1.08 ± 2.01	0.834^‡^
Colony-forming units (/100 cells)^*∗*^	3.13 ± 1.71	3.99 ± 1.52	0.428
Size of CFU (mm^2^)^*∗*^	9.91 ± 4.72	12.99 ± 3.26	0.263

^*∗*^Mean ± SD. ^†^Fisher's exact test. ^‡^Mann-Whitney *U* test.
